# Artificial Polysialic Acid Chains as Sialidase-Resistant Molecular-Anchors to Accumulate Particles on Neutrophil Extracellular Traps

**DOI:** 10.3389/fimmu.2017.01229

**Published:** 2017-09-29

**Authors:** Christina E. Galuska, Jan A. Dambon, Andrea Kühnle, Kim F. Bornhöfft, Gerlinde Prem, Kristina Zlatina, Thomas Lütteke, Sebastian P. Galuska

**Affiliations:** ^1^Institute of Reproductive Biology, Leibniz Institute for Farm Animal Biology (FBN), Dummerstorf, Germany; ^2^Faculty of Medicine, Institute of Biochemistry, Justus-Liebig-University, Giessen, Germany; ^3^Institute of Veterinary Physiology and Biochemistry, Justus-Liebig-University, Giessen, Germany

**Keywords:** neutrophil extracellular traps, polysialic acid, nanoparticle, histones, lipopolysaccharides

## Abstract

Neutrophils are involved in numerous immunological events. One mechanism of neutrophils to combat pathogens is the formation of neutrophil extracellular traps (NETs). Thereby, neutrophils use DNA fibers to form a meshwork of DNA and histones as well as several antimicrobial components to trap and kill invaders. However, the formation of NETs can lead to pathological conditions triggering among other things (e.g., sepsis or acute lung failure), which is mainly a consequence of the cytotoxic characteristics of accumulated extracellular histones. Interestingly, the carbohydrate polysialic acid represents a naturally occurring antagonist of the cytotoxic properties of extracellular histones. Inspired by polysialylated vesicles, we developed polysialylated nanoparticles. Since sialidases are frequently present in areas of NET formation, we protected the sensitive non-reducing end of these homopolymers. To this end, the terminal sialic acid residue of the non-reducing end was oxidized and directly coupled to nanoparticles. The covalently linked sialidase-resistant polysialic acid chains are still able to neutralize histone-mediated cytotoxicity and to initiate binding of these polysialylated particles to NET filaments. Furthermore, polysialylated fluorescent microspheres can be used as a bioanalytical tool to stain NET fibers. Thus, polySia chains might not only be a useful agent to reduce histone-mediated cytotoxicity but also an anchor to accumulate nanoparticles loaded with active substances in areas of NET formation.

## Introduction

More than 10 years ago, a novel defense mechanism of neutrophils was discovered. Besides the two classical ways—degranulation and phagocytosis—to fight against microbial invasion of pathogens, neutrophils are able to form a DNA-meshwork (1, 2). In these neutrophil extracellular traps (NETs), pathogens like bacteria and fungi are captured together with secreted antimicrobial components (3–6). Using the outlined NET mechanism, neutrophils can counteract a fast invasion of pathogens. Interestingly, the formation of NETs does not only take place during infection but also after insemination in the female reproductive tract (7). To escape the “capture mechanism” of neutrophils, seminal plasma includes DNases degrading the DNA fibers opening the way for spermatozoa to continue their journey to the oocyte. Furthermore, microcrystals like monosodium urate crystals, which accumulate for example in gout patients, induce the formation of NETs [for review, please see Ref. (8)].

Besides DNA, NETs consist of high concentrations of extracellular histones, which are toxic for endogenous cells before and after degradation of DNA scaffolds and, indeed, several diseases like fibrosis, sepsis, and autoimmune disorders as well as infertility are discussed to be triggered by these cytotoxic components of NETs (9–18). Thus, recently, possibilities have been in the focus of interest to inhibit and/or to neutralize the toxic effects of extracellular histones [for review, please see Ref. (19)].

For instance, antibodies against histones were tested demonstrating that antibody binding can reduce cytotoxicity (20). Nevertheless, it seems that sugar polymers like heparin (21) and polysialic acid (polySia) (20, 22–24)—a homo-polymer of α2,8-linked *N*-acetylneuraminic acid (Neu5Ac) residues—are more efficient (20). The disadvantage of all three applications is that in areas of inflammation, enzymes like proteases, heparinases, and sialidases are frequently present in high concentrations, which are able to degrade antibodies, heparin, or sialic acid polymers, respectively. Thus, universally applicable tools against the cytotoxicity of NETs have to be resistant against degradation or have to be applied repeatedly in high concentrations.

Inspired by the discovery of polysialylated vesicles in epididymis (23), we developed sialidase-resistant polysialylated nanoparticles in the present study. The coupled polySia chains were not only able to counteract the cytotoxic characteristics of extracellular histones but might also be a suitable “anchor” to enrich nanoparticles loaded with active substances (e.g., vaccines) on NETs and thus, on hotspots of inflammation.

## Experimental Section

### Materials

All reagents used were of analytical grade. Lipopolysaccharides (LPS) was removed from colominic acid (Gerbu, Heidelberg, Germany) for cell culture experiments using C18 cartridges (Thermo Fisher Scientific, Dreieich, Germany) according to the instructions. Colominic acid (polySia) is in the flow-through. LPS was detected using the Pierce™ LAL Chromogenic Endotoxin Quantitation Kit (Thermo Fisher Scientific).

### Fractionation of Sialic Acid Polymers

For native agarose gel separation experiments, 10 mg colominic acid (Gerbu) was separated and collected according to the degree of polymerization (DP) by anion exchange chromatography. Therefore, 4,5-methylene dioxybenzene (DMB)-labeled colominic acid was used to determine the retention time as described previously (24–26). The colominic acid standard was prepared using mild DMB labeling conditions as follows: 1 mg colominic acid (GERBU) was dissolved in 80 µl DMB reaction buffer [9 mM sodium hydrosulfite, 0.5 M β-mercaptoethanol, 20 mM trifluoroacetic acid (TFA), and 1.35 M DMB (Dojindo, Kumamoto, Japan)], and incubated overnight at 11°C (27, 28). The labeling was stopped by adding 20 µl 1 M NaOH. Sugar chains were separated on a DNAPac PAc-100 column (22 mm × 250 mm; 13 µm; Thermo, Idstein, Germany) by HPLC (Smartline System, Knauer, Berlin Germany). MilliQ water (E1) and 4 M ammonium acetate buffer (E2) were used as eluents at a flow rate of 2.5 ml/min. Following gradient was used for the separation: 0 min = 0% (v/v) E2, 20 min = 13% (v/v) E2, 30 min = 17% (v/v) E2, 45 min = 19% (v/v) E2, 85 min = 21% (v/v) E2, 110 min = 100% (v/v) E2. DMB-labeled polySia chains were detected using a fluorescence detector at 372 nm for excitation and 456 nm for emission.

### Determination of Chain Length Distribution by HPLC

In order to proof the chain length distribution, dried samples were treated by “mild” DMB analysis as described above. Samples were separated on an analytical DNAPac PAc-100 column (Thermo Fisher Scientific), 4 mm × 250 mm; 13 µm. MilliQ water (E1) and 4 M ammonium acetate buffer (E2) were used as eluents at a flow rate of 1 ml/min using the following gradient: 0 min = 0% E2; 5 min = 0% E2; 15 min = 8% E2; 20 min = 11% E2; 35 min = 14% E2; 55 min = 16% E2; 100 min = 20% E2; 130 min = 23% E2, and 131 min = 100% E2.

### Quantification of Neu5Ac by HPLC

For the quantification of Neu5Ac, sugar chains were hydrolyzed and labeled with DMB (29, 30). To this end, samples were dissolved in 0.2 M TFA for 4 h at 80°C. For DMB-labeling, hydrolyzates were dissolved in 80 µL DMB-reaction buffer and incubated at 55°C. After 2 h, reaction was stopped by adding 20 µL 0.2 N NaOH. Resulting DMB-Neu5Ac molecules were analyzed on a Superspher 100 C-18 column (250 mm × 40 mm, Merck-Hitachi, Darmstadt, Germany) at 40°C by HPLC (Smartline System) as described before in detail (31, 32).

### Agarose Gel-Electrophoresis

For native agarose gel separation histones (Sigma-Aldrich, Steinheim, Germany), catalase from bovine liver (Serva, Heidelberg, Germany) and aldolase from rabbit muscle (Serva) was used. All proteins were incubated with fractionized or original colominic acid in 50 mM Tris for 1 h at 30°C on a shaker. After incubation, samples were loaded on a 2% agarose gel (w/v) (peqGOLD Universal Agarose, peqLab, Erlangen, Germany) in 500 mM Tris/HCL, 160 mM boric acid, 1 M urea (pH 8.5), and running buffer [90 mM Tris/HCL, 90 mM boric acid (pH 8.5)] was added. The electrophoresis was performed at 80 V (constant voltage) for approximately 4 h, cooled by icepacks [modified from Ref. (33, 34)]. Fixation [45% methanol (v/v); 10% acetic acid (v/v)] of proteins in the gel was done overnight. For coomassie blue staining, the gel was stained with roti-blue (Carl Roth, Karlsruhe, Germany) according to manufacturer’s instructions.

For DNA agarose gel-electrophoresis, colominic acid, histones, and 100 bp Plus Blue DNA ladder (GeneOn, Ludwigshafen am Rhein, Germany) were used. DNA ladder and colominic acid as well as DNA ladder and histones were incubated for 1 h at room temperature. After the incubation, the samples were loaded onto a 2% agarose gel (w/v) with SyBR Gold nucleic acid gel stain (Thermo Fisher Scientific). The electrophoresis was performed at 70 V, 500 mA, and 150 W for 1.5 h in 1× Rotiphorese TAE-buffer (Carl Roth). Imaging was performed by the Herolab transluminator (Herolab, Wiesloch, Germany).

### Modeling

Molecular docking experiments were carried out with AutoDock3 [DOI: 10.1002/(SICI)1096-987X(19981115)19:14 < 1639:AID-JCC10 > 3.0.CO;2-B] *via* the YASARA interface. A 3D structure model of the Neu5Ac-(α2,8)-Neu5Ac disaccharide was created with YASARA (PMID: 24996895) and docked to the histone complex of Protein Data Bank (PDB) entry 3wa9 (PMID: 24311584), from which DNA had been removed before docking (data not shown). The same PDB entry, again without DNA fragments, was also used in a molecular dynamics (MD) simulation. The 3D structure of a polySia chain with 20 residues was modeled with YASARA; four copies of this model have been placed in a distance of 8–15 Å to the histone, and a water box has been added. The MD simulation has been performed with YASARA and AMBER03 force field [PMID: 14531054] for 6 ns.

### Oxidation of Colominic Acid

Colominic acid was activated by selective periodate oxidation as described by Jennings and Lugowski (35). Freshly prepared 0.1 M sodium metaperiodate was mixed with 10 mg colominic acid and the solution stirred at 4°C for 4 h in the dark. In order to stop the reaction, a twofold volume of ethylene glycol was added to the reaction mixture and left to stir at 20°C for further 30 min. Finally, the solution was dialyzed for 24 h against 25 mM ammonium bicarbonate (pH 7.4) at 20°C and subsequently dried.

### Polysialylated Latex Beads

After washing, aliphatic amine latex beads (0.1 µm, Thermo Fisher Scientific) were resuspended in PBS using an ultrasonic homogenizer. Beads were then polysialylated by reductive amination adding oxidized colominic acid and 50 mM sodium cyanobrohydride in PBS (pH 7.4). After incubation in the dark at 4°C overnight, beads were extensively washed and resuspended in RPMI medium for further experiments.

### Sialidase Digest of Polysialylated Fluorescence Beads

An aliquot of the polysialylated beads was diluted in sodium acetate buffer (50 mM, pH 5.5). A sialidase mixture (2 mU sialidase from *Vibrio cholerae* (Sigma-Aldrich), 100 U sialidase from *Clostridium perfringens* (New England BioLabs, Frankfurt, Germany)) was added. After incubation of the digest at 37°C overnight, the mixture was centrifuged at 14,000 rpm. The supernatant as well as the pellet were used for Neu5Ac quantification as described above.

### Polysialylated Fluorescence Beads

Particles were polysialylated by reductive amination as described by Wu et al. (36). The oxidized colominic acid chains were covalently linked to the particles (fluorescence amine modified latex beads, Sigma-Aldrich) in the presence of sodium cyanoborohydride in PBS (pH 7.4). The solution was magnetically stirred at 37°C for 48 h in the dark (37, 38). The modified beads were extensively washed with PBS and used for NET-visualization and cytotoxicity assays.

### Cell Culture Experiments

5B8 cells were cultured as described earlier in detail (24). 30,000 cells per well were seeded in 96-well plates in RPMI medium (Gibco, Darmstadt, Germany) for histone assays. The cells were incubated for 90 min with histones and/or oxidized as well as native polySia or polysialylated nanoparticles. The cytotoxicity was determined with the lactate dehydrogenase cytotoxicity assay (BioVision, Milpitas, CA, USA).

### NET-Binding Assay

Neutrophil granulocytes were isolated and NETs induced as described by Saffarzadeh et al. (20). 70,000 cells/well were dissolved in RPMI (Thermo Fisher Scientific) with 1% penicillin/streptomycin (Thermo Fisher Scientific) and 1% FBS (Thermo Fisher Scientific) and incubated in a CO_2_ incubator at 37°C for 1 h. For the induction of NETosis, 20 nM phorbol myristate acetate (PMA) was added before continuing incubation in a CO_2_ incubator at 37°C for 4 h. Finally, the cells were fixated on the cover slips with 4% paraformaldehyde for 30 min at 37°C. Fixated cells were washed multiple times with PBS (pH 7.4) after being removed from the 24-well plates. For permeabilization, the cells were incubated with 0.5% Triton X-100 for 1 min at 20°C and washed again with PBS. Samples were incubated with different concentration of polysialylated beads for 1 h at 37°C and mildly washed again with PBS to minimize unspecific interactions. Controls included unconjugated beads. After blocking with 2% IgG-free albumin (Carl Roth) for 1 h, samples were incubated with an mAb against neutrophil elastase (M-18; Santa Cruz Biotechnology, Heidelberg, Germany) followed by a secondary FITC-conjugated antibody (Santa Cruz Biotechnology). Vectashield antifade mounting medium with DAPI (Vector Laboratories Inc., Burlingame, CA, USA) was used for DNA detection. For image quantification of red fluorescent polysialylated particles in NET fibers, the red RGB channel was separated with Adobe Photoshop CS4 EXTENDED (11.0, Adobe Systems Software, Dublin 24, Ireland) and stained areas were calculated with CellProfiler (39).

In addition, experiments were performed without a paraformaldehyde fixation before the application of the polysialylated fluorescence beads. Therefore, 30,000 cells per well were dissolved in RPMI (Thermo Fisher Scientific) with 1% penicillin/streptomycin (Thermo Fisher Scientific) and 1% FBS (Thermo Fisher Scientific) and incubated in a CO_2_ incubator (5%) at 37°C for 1 h. For the induction of NETosis, 20 nM PMA (Sigma-Aldrich) or 2 µg/mL LPS from *Pseudomonas aeruginosa* (Sigma-Aldrich) was added and cells were further incubated at 37°C and 5% CO_2_ for 4 h. After three times of washing with RPMI, in order to remove the remaining LPS or PMA, cells were incubated with polysialylated beads (325 µg/mL) for 30 min at 37°C. Subsequently, cells were fixed with 4% paraformaldehyde for 30 min and incubated with 0.5% Triton X-100 (Sigma-Aldrich) for 1 min and washed again with PBS followed by blocking with 2% IgG-free BSA (Carl Roth) at 37°C for 30 min. Antibody against neutrophil elastase (M-18) was incubated overnight. After multiple washing steps with PBS, the secondary FITC-conjugated antibody was incubated for 1 h, prior DAPI (Carl Roth) counterstaining and mounting.

### Statistical Analysis

Data sets were analyzed by GraphPad Prism 7 software using one-way analysis of variance (ANOVA) with Tukey posttests for multiple comparisons. Differences were considered statistically significant at *p* < 0.05. Significant differences are given: *p < 0.05; **p < 0.01; ***p < 0.001; ****p < 0.0001.

## Results

### PolySia Chains Interact with Histones

In previous studies, we already speculate that polySia might acts as a molecular anchor to accumulate polysialylated vesicles and immune cells on NETs (22, 23). Based on this hypothesis, we wanted to develop artificial polysialylated nanoparticles for biomedical applications to decrease NET-mediated cytotoxicity and to enrich nanoparticles *via* polySia chains on NET-fibers.

**Figure 1:**
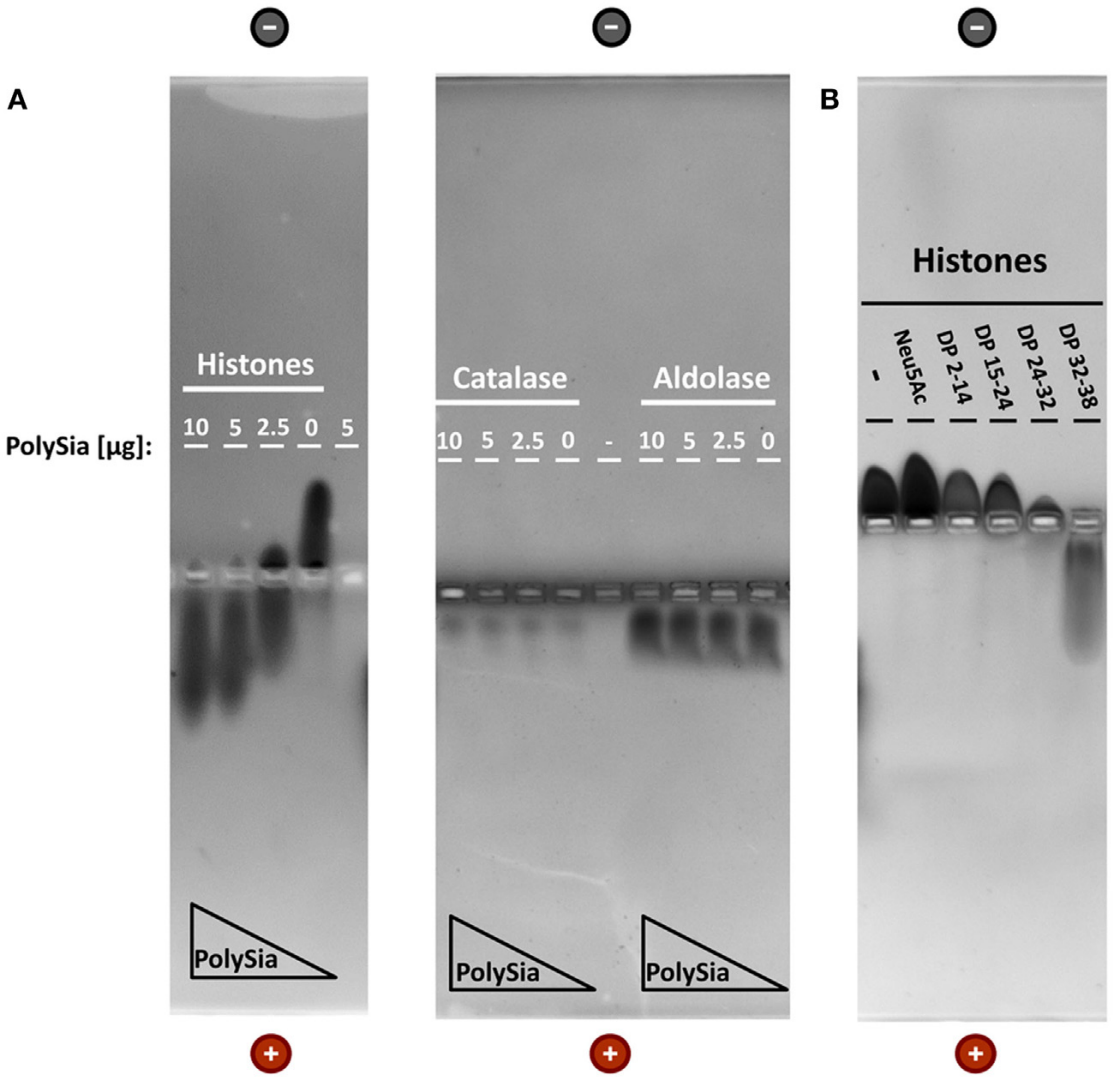
PolySia interact with histones. Non-covalent binding assays of histones and polySia were applied using native gel electrophoresis. **(A)** Proteins (5 µg) were preincubated with different concentrations of polySia (mixture of different chain lengths). Due to the interaction with highly negatively charged polySia chains, histones–polySia complexes migrate together to the positive pole. Catalase and aldolase were used as negative controls. **(B)** In addition, histones (10 µg) were incubated with altered chain lengths of polySia (all 5 µg). Proteins were stained with Coomassie Blue.

An accumulation of such polySia-coated nanoparticles, however, would require an interaction between histones and polySia. To investigate the binding properties between polySia and histone complexes, native gel electrophoresis was applied. To this end, histones were incubated with increasing concentrations of polySia. As shown in Figure 1A, the migration characteristics of histones are directly influenced by polySia resulting in a shift to the anode. The movement of control proteins like catalase and aldolase was not influenced by polySia indicating that a specific binding is necessary to influence the migration characteristics.

In order to monitor, if polySia can also interact with DNA, polySia was incubated with DNA and separated *via* agarose gel electrophoresis (Figure S1 in Supplementary Material). In comparison to the “DNA only” sample, the migration characteristics of DNA in the presence of polySia did not change. However, histones lead, as expected, to a significant impaired migration capacity of DNA. Thus, polySia chains can only bind histones, but not DNA.

Interestingly, the interaction between histones and polySia depends on the DP (Figure 1B). Fractionized chains with DPs between 24–32 and 32–38 extensively influence the migration distance. However, first, but only small migration effects could also be observed using a fraction with DP 15–24. The different groups of chain length were prepared as described in Ref. (24) and the chain lengths of all collected fractions were tested using “mild” DMB analysis (Figure S2 in Supplementary Material).

**Figure 2:**
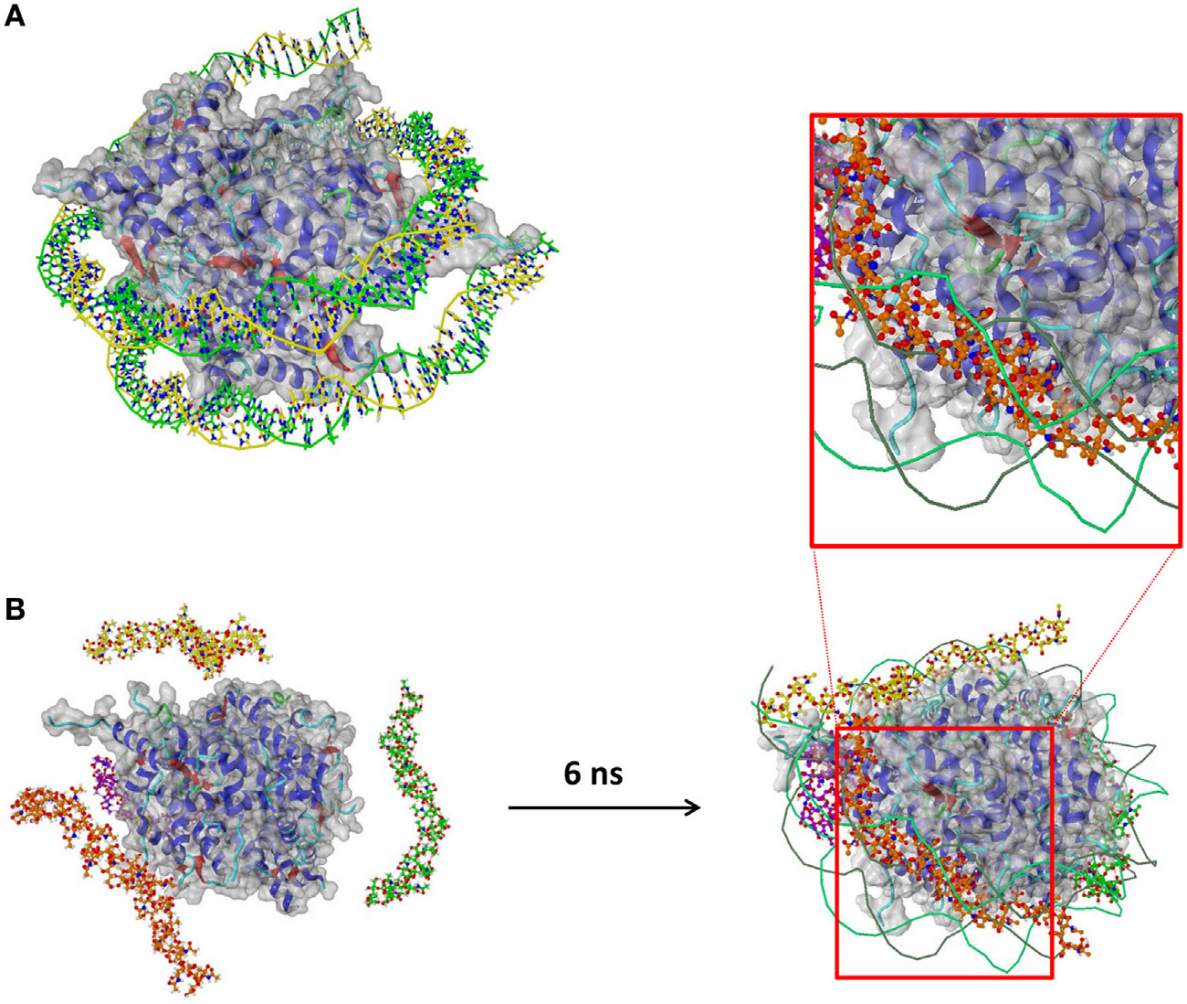
Molecular dynamics (MD) simulation of polySia chains and histone. **(A)** The histone part of this complex has been used as receptor in a docking study and an MD simulation after removing the DNA fragments (Histone–DNA complex, Protein Data Bank entry 3wa9). **(B)** Four chains of 20 sialic acids each have been placed close to the histone and put into a water box (water not shown). After 6 ns, the polySia chains have bound to the histone and moved to the areas that bind to DNA.

In addition, the molecular basis of the interaction between polySia chains and histones has been investigated by a docking study and a MD simulation. A 3D structure of a histone DNA complex, taken from the PDB, has been used as a receptor (Figure 2A). Superimposition of docking results and the DNA fragments that bind to the histone complex in the original PDB entry reveals that the majorities of the top 25 docking poses are placed at the DNA-binding areas of histones. To investigate the binding of longer polySia chains, each of four chains of 20 sialic acids have been placed close to the histone in a water box as a starting structure of an MD simulation. We used a chain length of 20 sialic acid residues, since first protective effects against the cytotoxicity of histones were previously observed in this size range (24). After 6 ns, all four polySia chains have approached the histones and moved to comparable areas that bind to DNA in the original PDB structure, independent of the orientation of the chains at the beginning of the simulation (Figure 2B).

This indicates that polySia chains are able to bind histone complexes in a way comparable with DNA.

### Not Only Natural polySia But Also Oxidized PolySia Chains Counteract Histone-Mediated Cytotoxicity

**Figure 3:**
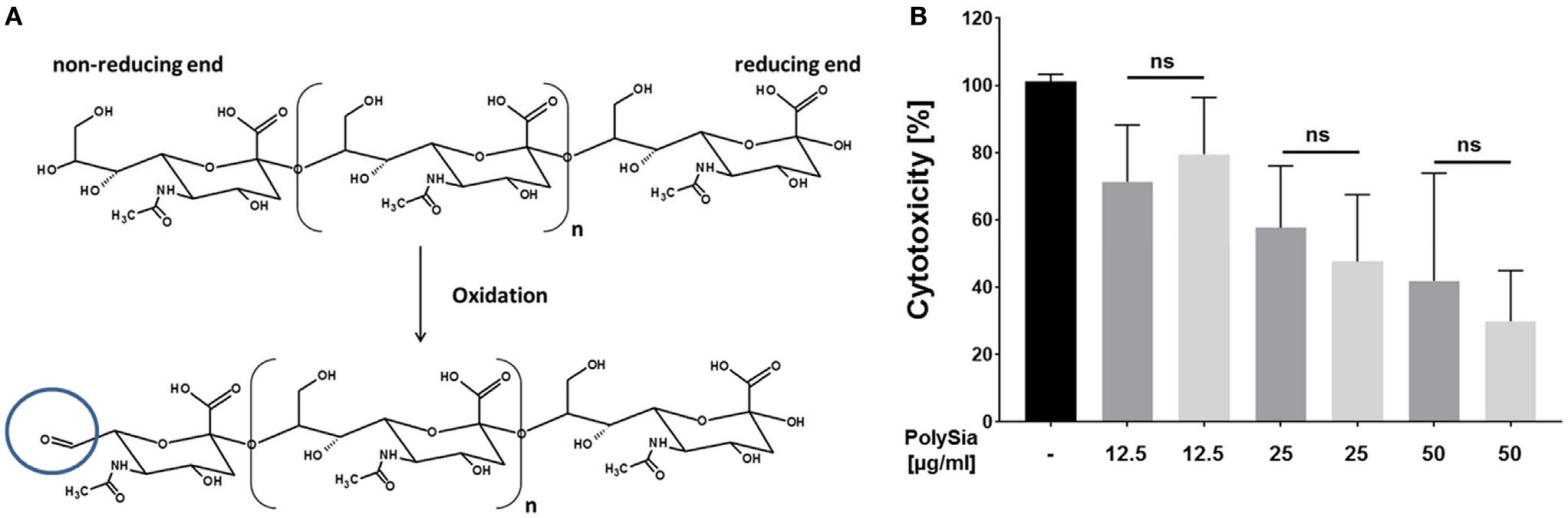
Oxidized polySia counteract the cytotoxicity of histones. **(A)** Illustration of the oxidation of polySia resulting in the formation of a C7 body at the non-reducing end. **(B)** Native and oxidized polySia were tested for their ability to compensate histone-mediated cytotoxicity. Cells were treated with histones (60 µg/ml) and the cytotoxicity was determined (black). In parallel, the cytotoxicity was determined in the presence of native (dark gray) and oxidized polySia (light gray). 100% cytotoxicity was set for histone-treated cells. All values are means of three independent experiments. The statistical evaluation was performed by one-way analysis of variance analysis. ns, not significant; *p < 0.05; **p < 0.01; ***p < 0.001.

Since during several physiological and pathophysiological events, which induce NET formation, sialidases are also present (40–43), a protection against degradation would be of great advantage. Mammalian as well as bacterial sialidases require a free non-reducing end for a stepwise release of terminal sialic acid residues. In order to mask the non-reducing end, polySia was oxidized by sodium metaperiodate. The transformation of the terminal sialic acid on the non-reducing end into a C7 residue results in the formation of an aldehyde group at C7 (Figure 3A). Resulting oxidized polySia chains were tested for their ability to counteract histone-mediated cytotoxicity. As shown in Figure 3B, oxidized as well as native sialic acid polymers show comparable characteristics to decrease histone-mediated cytotoxicity. The results are comparable with the MD simulation, which suggests that the reducing- as well as non-reducing ends of the chains are not directly involved in histone binding (Figure 2B). Furthermore, it must be noted that all commercial available colominic acid samples (e.g., Sigma-Aldrich, Gerbu), which we tested, were contaminated with lipopolysaccharides (LPS) (~ 3% w/w). For this reason, LPS was removed using C18 cartridges for the described experiment.

### Nanoparticles with Sialidase-Resistant PolySia Chains Can Counteract Histone-Mediated Cytotoxicity

**Figure 4:**
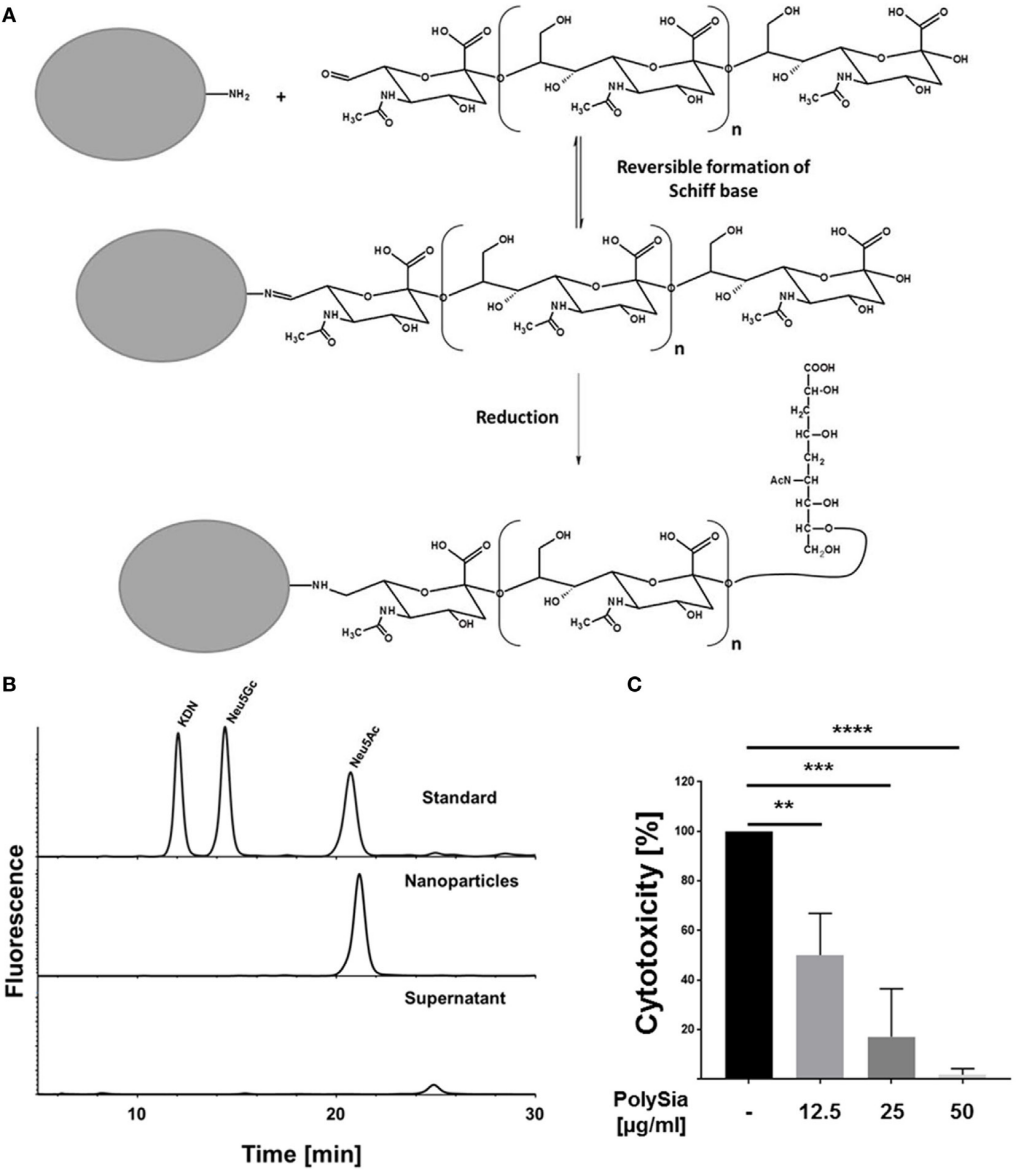
Polysialylated nanoparticles counteract histone-mediated cytotoxicity. **(A)** Illustration of the chemical polysialylation on amino modified particles. **(B)** The sialidases sensitivity of the oxidized polySia chains on nanoparticles were tested using bacterial sialidases. After enzymatic treatment, beads were centrifuged and supernatant as well as the bead-pellet were checked for the presence of Neu5Ac by DMB-HPLC analysis. A sialic acid standard panel (KDN, Neu5GC, and Neu5Ac) was used to determine the retention time of Neu5Ac. **(C)** In addition, polysialylated nanoparticles were tested for their capability to compensate histone-mediated cytotoxicity. Cells were treated with histones (60 µg/ml) and the cytotoxicity was determined. In parallel, the cytotoxicity was determined in the presence of different concentration of polysialylated nanoparticles. 100% cytotoxicity was set for histone-treated cells. All values are means of three independent experiments. The statistical evaluation was performed by one-way analysis of variance analysis. ns, not significant; *p < 0.05; **p < 0.01; ***p < 0.001; ****p < 0.0001.

In a next step, oxidized polySia chains were coupled with the oxidized non-reducing end to latex beads (nanoparticle diameter ~100 nm). To this end, the unstable Schiff base—formed during the reaction between the aldehyde group of polySia and the primary amide group on nanoparticles—was reduced (Figure 4A). The chemical coupling was tested by “mild” DMB-labeling after several washing steps, demonstrating that polySia chains were attached to nanoparticles (data not shown). Furthermore, nanoparticles were incubated with sialidases to test their resistance against enzymatic degradation. Subsequently, nanoparticles were centrifuged after sialidase treatment and the supernatant was examined for released sialic acid residues using HPLC analysis after hydrolysis and fluorescence labeling. In parallel, polySia-beads were analyzed. Whereas no sialic acid residues were detectable in the supernatant, analysis of polysialylated nanoparticles led to a strong DMB-Neu5Ac signal (Figure 4B). Accordingly, sialidases were not able to degrade the covalently coupled polySia chains.

Furthermore, polysialylated nanoparticles were tested for their capability to protect cells against extracellular histones. In a first set of experiments, however, polysialylated beads were tested for its own cytotoxicity. The polysialylated nanoparticles showed no toxicity and displayed positive effects (between 20 and 30%) on the determined cytotoxicity values in comparison to untreated cells (Figure S3 in Supplementary Material). In addition, cells were treated with histones in the presence of the polysialylated nanoparticles. As indicated in Figure 4C, polysialylated nanoparticles neutralize the cytotoxic effects of extracellular histones in a concentration-dependent manner.

Accordingly, the experiments demonstrated that due to oxidation and coupling of polySia to nanoparticles, the non-reducing end is protected against degradation by sialidases without a loss of its ability to counteract histone-mediated cytotoxicity.

### Polysialylated Particles Accumulate on NET Fibers

Finally, we wanted to investigate if polySia can not only counteract the cytotoxic characteristics of extracellular histones, but also act as an anchor to accumulate “cargos” on NETs. For this part of the study, oxidized polySia chains were coupled to fluorescent beads.

**Figure 5:**
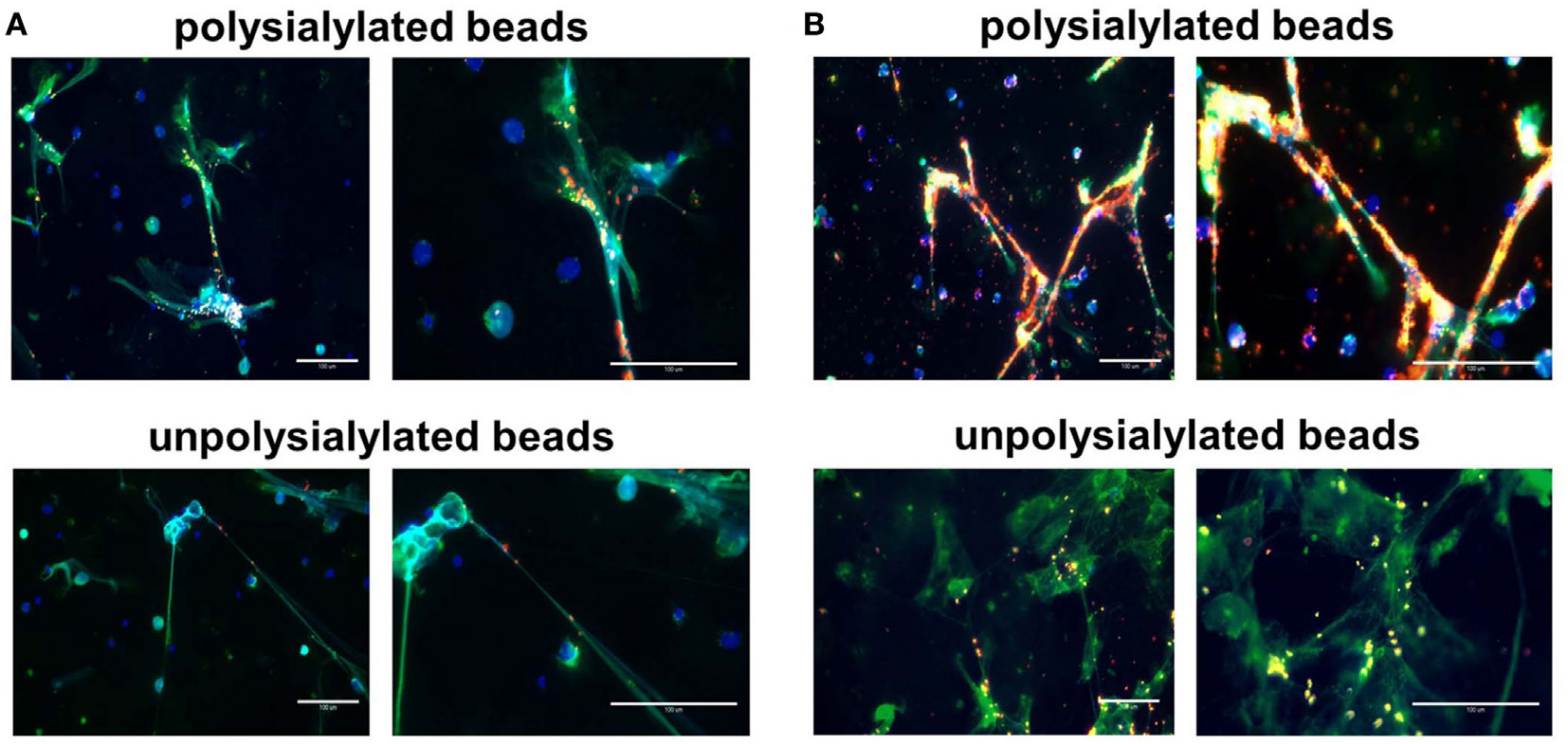
Polysialylated nanoparticles accumulate on NET. Polysialylated as well as unpolysialylated fluorescence beads (in red) were incubated with neutrophils after phorbol myristate acetate treatment. **(A)** 32.5 µg beads/ml; **(B)** 325 µg beads/ml. NET was visualized by DAPI and an antibody against neutrophil elastase. Scale bar: 100 µm.

Successively, neutrophils were enriched and NET formation was induced by PMA. NETs were visualized by neutrophil elastase detection and DAPI staining after fixation using paraformaldehyde. As shown in Figure 5, polysialylated fluorescence beads strongly accumulate on NETs. Approximately 97% of the determined red fluorescence signals were associated with NET filaments in both applied concentration of polysialylated fluorescence beads. Unpolysialylated beads show no specific binding to NETs. In contrast to NET fibers, unstimulated neutrophils were not labeled *via* polysialylated beads (Figure S4 in Supplementary Material).

In addition, polysialylated fluorescence beads were applied to unfixed neutrophils after PMA or LPS stimulation. As shown in Figure S5 in Supplementary Material, polysialylated beads accumulate also on native NETs after PMA as well as LPS stimulation. Along with the obtained results using unstimulated neutrophils after paraformaldehyde fixation, unstimulated native neutrophils showed no binding characteristics for polysialylated beads.

Thus, the results demonstrate that the generated bioconjugates are resistant against sialidases, inhibit the cytotoxic effect of extracellular histones, and can be used to enrich particles on NETs.

## Discussion

Neutrophil extracellular traps are formed during several biological events. During this process, neutrophils release a mixture of DNA and histones building up a “ball of yarn” consisting of DNA-fibers and several antimicrobial factors. In addition to neutrophils, several other cells, like eosinophils (44), monocytes/macrophages (45), and mast cells (46), produce these fibers of extracellular chromatin. However, NET is the best studied extracellular trap so far. Interestingly, NETs are discussed to be negative key modulators during pathological events like sepsis, asthma, and numerous other diseases (18, 19). Especially in lung, therapies-targeting NETs are under present investigation using anti-histone antibodies, protease inhibitors, and DNases to counteract the “dark side” of NETs (47).

Intriguingly, polySia seems to be frequently present in areas where neutrophils form NETs. For instance, lung epithelial cells secrete polySia during inflammation and polySia is present in ejaculates of mammals (22, 23). Thus, polySia might be a naturally occurring antagonist against histone-mediated cytotoxicity. For this reason, we wanted to develop polySia-based strategies to neutralize the negative aspects of NETs. Together, with our observation that vesicles can be polysialylated (22), we were inspired to develop polysialylated particles as a possibility to accumulate nanoparticles of interest on these hot spots of inflammation. As a fundamental requirement, however, polySia does not only have to inactivate the cytotoxicity of extracellular histones but also needs to build complexes with histones. Our modeling study suggests that a direct interaction between histones and polySia consisting of more than 20 Neu5Ac residues takes place. The results are in line with our previous study demonstrating that only polySia chains with more than 20 sialic acid residues start to reduce the cytotoxicity of extracellular histones (24). Thus, the basic prerequisite for an accumulation of polysialylated “cargos” and simultaneous inactivation of cytotoxic histones is given.

**Figure 6:**
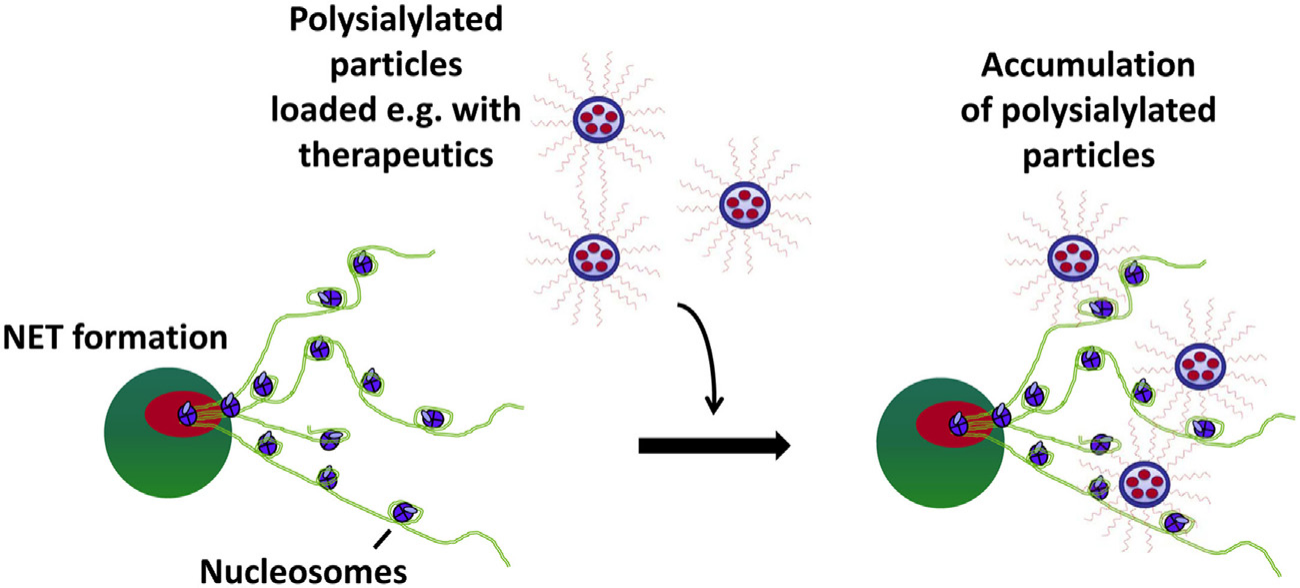
Polysialylated nanoparticles accumulate on neutrophil extracellular trap (NET). Polysialylated beads are able to bind and accumulate at histones/DNA-fibers *via* their polySia chains.

However, it has to be considered that several pathogens and also body’s own cells like spermatozoa are able to secret sialidases during inflammatory conditions (40, 43, 48), which would inactivate a polySia-based system due to the destruction of these sugar chains. Consequently, sialidase-resistant polySia chains would be favorably. Since bacterial as well as mammalian sialidases require a free non-reducing end, this weak spot has to be protected. We oxidized the non-reducing end, as it is not involved in histone-binding, and the resulting aldehyde groups were used to couple polySia chains with the non-reducing end on nanoparticles bearing primary amino groups. Consequently, an enzymatic degradation by bacterial and eukaryotic sialidases is no longer possible, which was verified by bacterial sialidases. The modified polySia chains were still able to neutralize the cytotoxic capability of extracellular histones. Moreover, using polysialylated fluorescent beads, a binding *via* these oxidized Neu5Ac-polymers on NETs was demonstrated. Thus, polySia can be used as an anchor to accumulate nanoparticles on hotspots of inflammation (Figure 6). However, it has to be kept in mind that several types of nanoparticles are discussed to induce NETosis (49–53). Thus, the selected nanoparticles have to be tested for their capacity to induce NETosis before *in vivo* application.

Furthermore, polysialylated nanoparticles may aggregate DNA filaments during NET formation, since one polysialylated bead can theoretically bind more than one nucleosome. Whether polysialylated beads induce the aggregation of NET fibers during the formation of NET in reality as well as the potential consequences will be studied in future.

In addition to the outlined therapeutic application, polysialylated fluorescent beads could be a useful tool to visualize NETs and other extracellular traps due to their binding properties for heterochromatin and represent, consequently, a further biochemical method to study NET formation.

In conclusion, our data revealed that sialidases-resistant polysialylated nanoparticles might represent a useful instrument to counteract histone-mediated cytotoxicity and moreover, to enrich “cargos” loaded with active substances, which might be released in a configurable temporal path in areas of NET formation, to trigger inflammatory processes.

## Ethics Statement

This study was carried out in accordance with the recommendations of ethics committee of the Medical Faculty JLU Giessen with written informed consent from all subjects. All subjects gave written informed consent in accordance with the Declaration of Helsinki. The protocol was approved by the ethics committee of the Medical Faculty JLU Giessen (reference number 05/00).

## Author Contributions

CG, JD, KZ, AK, KB, and GP performed experiments, analyzed the data, and wrote the experimental section. TL prepared the model. SG wrote also the experimental part in addition to all other sections of the manuscript. All authors reviewed the results as well as the manuscript and approved the final version of the manuscript.

## Conflict of Interest Statement

The authors declare that the research was conducted in the absence of any commercial or financial relationships that could be construed as a potential conflict of interest.

## Abbreviations

DMB, 4,5-methylene dioxybenzene; DP, degree of polymerization; MD, molecular dynamics; NETs, neutrophil extracellular traps; Neu5Ac, N-acetylneuraminic acid; PMA, phorbol myristate acetate; polySia, polysialic acid; PDB, Protein Data Bank; TFA, trifluoroacetic acid.
