# Supplementary material for: Artificial Polysialic Acid Chains as Sialidase-Resistant Molecular-Anchors to Accumulate Particles on Neutrophil Extracellular Traps
Source: Front Immunol. 2017 Sep 29;8:1229. doi: 10.3389/fimmu.2017.01229 (PMC5626807; doi:10.3389/fimmu.2017.01229)
Supplement: Supplementary file 1 [file Image_1.PDF]

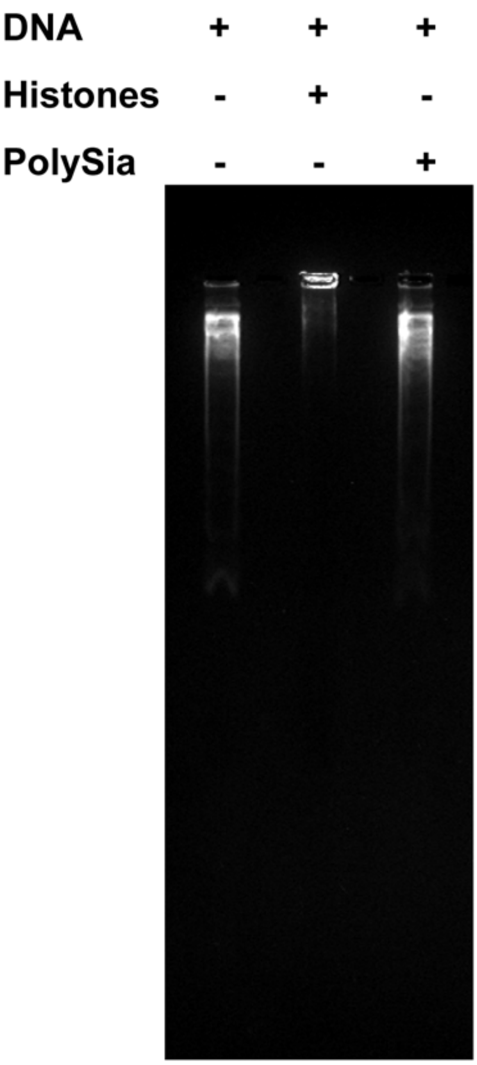

Figure S1. DNA interact with histones but not with polySia. DNA (0.25 µg) was directly separated by agarose gel-electrophoresis or after incubation with histones (2.5 µg) or polySia (2.5 µg).

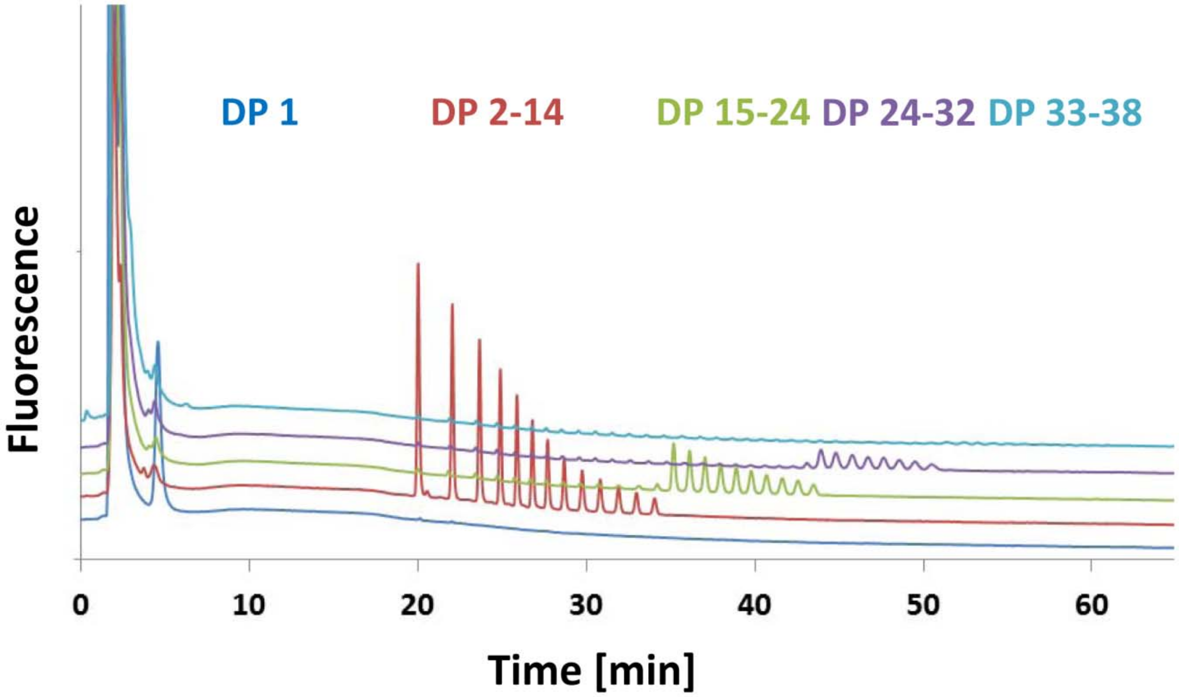

Figure S2. Fractionation of polySia. Defined chain lengths of unlabeled sialic acid polymers were purified. The distinct chain lengths of all fractions were controlled by rechromatography after DMB labeling. Neu5Ac content of all collected fractions was determined by DMB-HPLC analysis (data not shown).

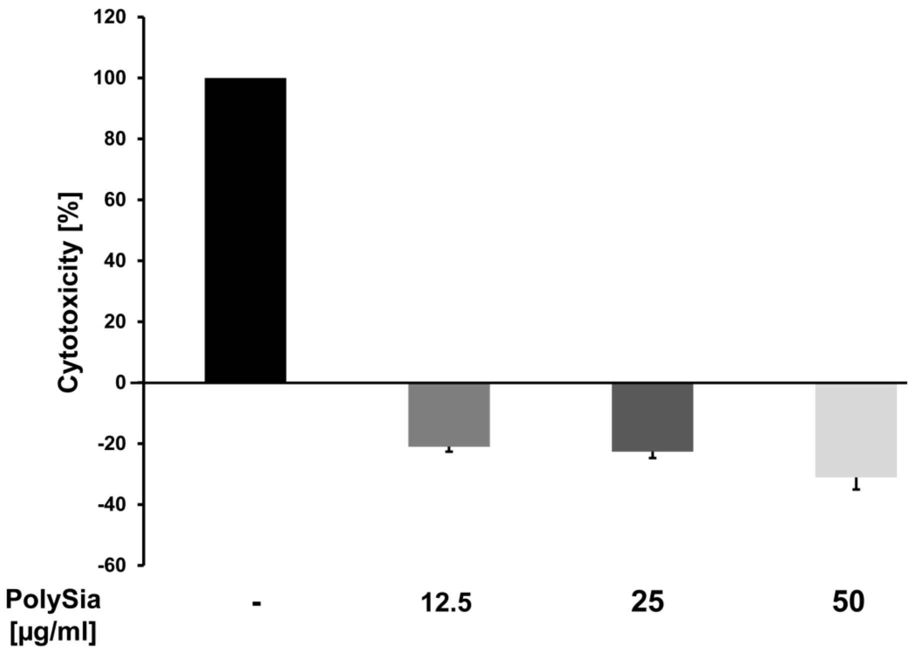

Figure S3. Polysialylated nanoparticles displayed no toxicity. Polysialylated nanoparticles were tested for their cytotoxicity. Cells were treated with histones (60 µg/ml) and the cytotoxicity was determined. In addition, the cytotoxicity was determined in the presence of different concentrations of polysialylated nanoparticles without histones. 100% cytotoxicity was set for histone treated cells. All values are means of 3 independent experiments.

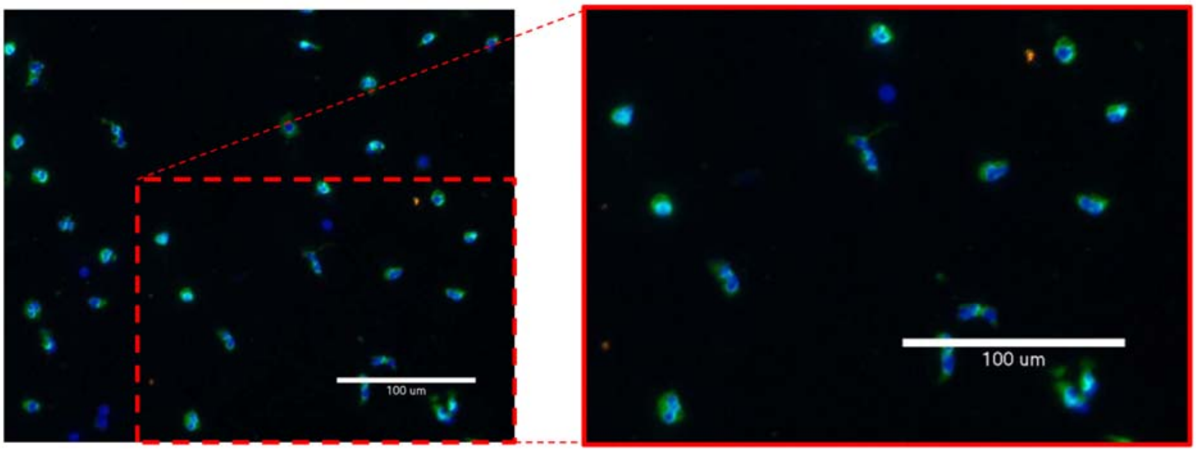

Figure S4. Polysialylated fluorescence beads do not accumulate on unstimulated neutrophils. Polysialylated red fluorescence beads (32.5 µg beads/ml) were incubated with neutrophils. DNA was visualized by DAPI and an antibody against neutrophil elastase was used. Scale bar: 100 µm.

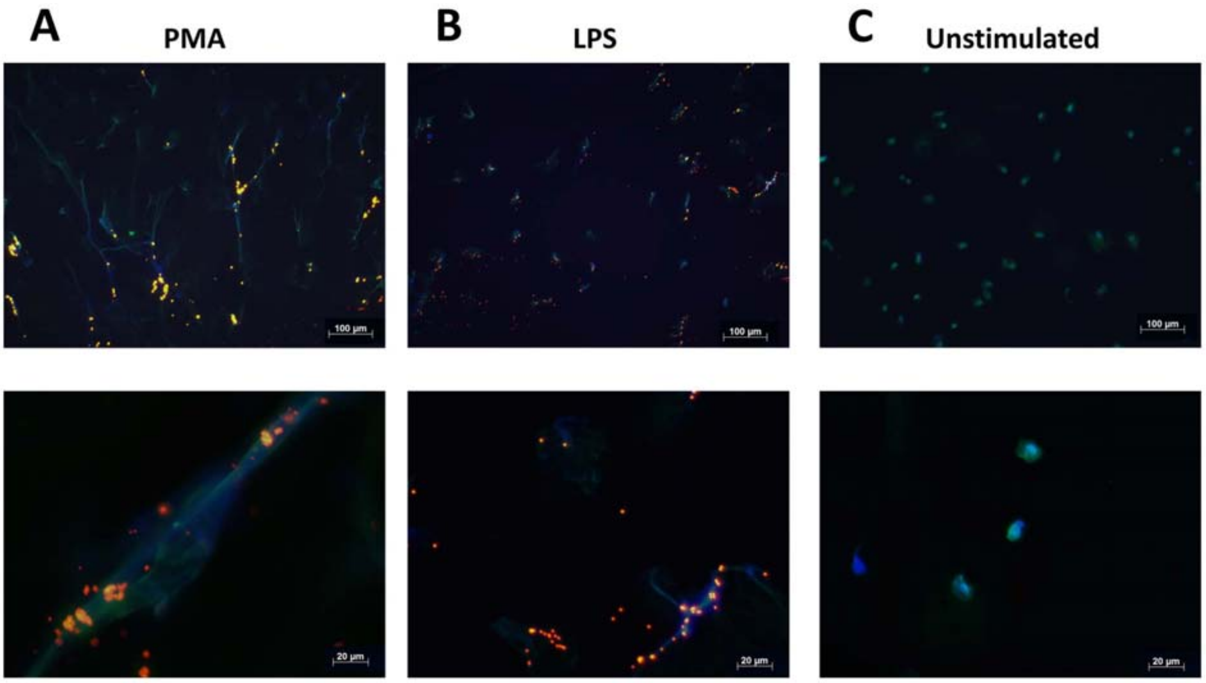

Figure S5. Polysialylated fluorescence beads bind unfixed NETs. Polysialylated red fluorescence beads (325 µg beads/ml) were incubated with neutrophils after PMA or LPS treatment. In addition, unstimulated neutrophils were used. DNA was visualized by DAPI and an antibody against neutrophil elastase was used. Scale bars: 25 and 100 µm.
